# A quantitative, high-throughput method identifies protein–glycan interactions via mass spectrometry

**DOI:** 10.1038/s42003-019-0507-2

**Published:** 2019-07-22

**Authors:** Pavel I. Kitov, Elena N. Kitova, Ling Han, Zhixiong Li, Jaesoo Jung, Emily Rodrigues, Carmanah D. Hunter, Christopher W. Cairo, Matthew S. Macauley, John S. Klassen

**Affiliations:** 1 Alberta Glycomics Centre and Department of Chemistry, University of Alberta, Edmonton, AB T6G 2G2 Canada; 2 Department of Medical Microbiology and Immunology, University of Alberta, Edmonton, AB T6G 2E1 Canada

**Keywords:** Mass spectrometry, High-throughput screening

## Abstract

Glycan binding by glycan-binding proteins and processing by carbohydrate-active enzymes is implicated in physiological and pathophysiological processes. Comprehensive mapping of glycan interactions is essential to understanding of glycan-mediated biology and can guide the development of new diagnostics and therapeutics. Here, we introduce the competitive universal proxy receptor assay (CUPRA), which combines electrospray ionization mass spectrometry, competitive binding and heterobifunctional glycan-based ligands to give a quantitative high-throughput method for screening glycan libraries against glycan-binding and glycan-processing proteins. Application of the assay to human (siglec-2), plant (*Sambucus nigra* and *Maackia amurensis* lectins) and bacterial (cholera toxin, and family 51 carbohydrate binding module) proteins allowed for the identification of ligands with affinities (*K*_d_) ≤ 1 mM. The assay is unprecedentedly versatile and can be applied to natural libraries and, when implemented in a time-resolved manner, provides a quantitative measure of the activities and substrate specificity of carbohydrate-active enzymes.

## Abstract (web summary)

Kitov et al. present the competitive universal proxy receptor assay (CUPRA) that provides a quantitative and high-throughput method for screening glycan libraries against glycan-binding and glycan–processing proteins. This assay measures the activity and substrate specificity of lectins and carbohydrate-active enzymes.

## Introduction

Glycan binding by glycan-binding proteins (GBPs) and processing by Carbohydrate-Active enZymes (CAZymes) is implicated in almost all physiological and pathophysiological processes, including cell recognition and signaling, the immune response, bacterial and viral infections, cancer metastasis, metabolic, autoimmune and neurodegenerative diseases^1^. Comprehensive mapping of glycan interactions with GBPs and CAZymes is essential for a thorough understanding of glycan-mediated biology and guides the development of new diagnostics and therapeutics for a wide range of diseases^2,3^.

Glycan microarrays, in which oligosaccharides are immobilized through a linker on a solid surface, is the dominant technology for high-throughput screening of oligosaccharides libraries, both synthetic and natural^4^. Despite their extensive use for establishing glycan-binding specificities of GBPs, glycan array screening has a number of well-known limitations. The assay is not quantitative, exhibits artefacts associated with glycan modification, immobilization and protein labeling (with a fluorophore), and is prone to false negatives, particularly for low affinity interactions, due to the necessary washing steps^5^. Consequently, the correlation between glycan array data and trends in oligosaccharide affinities may be poor^6^. An alternative to glycan microarrays is catch-and-release electrospray ionization mass spectrometry (ESI-MS), a label- and immobilization-free assay capable of simultaneously screening hundreds of oligosaccharides against GBPs^7^. The method, which is based on the detection of charged oligosaccharides released from gaseous GBP ions produced by ESI performed on an aqueous solution of GBP and oligosaccharide library, is able to detect very low affinity (*K*_d_~1 mM) interactions^7^. Although catch-and-release ESI-MS is rapid and consumes small amounts of sample, it provides, at best, an approximate ranking of affinities.

**Fig. 1:**
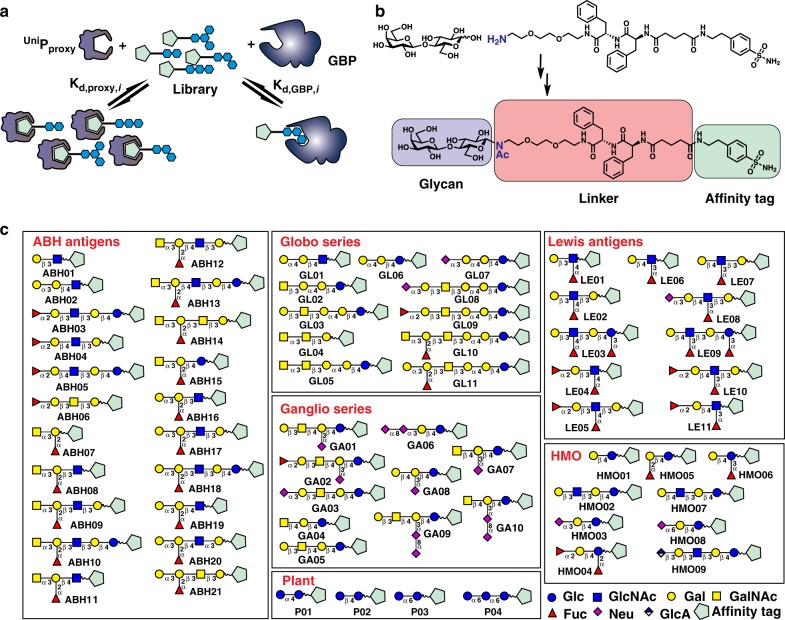
Overview of CUPRA library screening. **a** Competitive binding is the basis of CUPRA library screening. The universal proxy protein (^Uni^P_proxy_) binds to an affinity tag present in all members of the library of modified oligosaccharides (OS^mod^); ligand binding (to GBP) is identified and quantified from the depletion of ^Uni^P_proxy_–OS^mod^ complexes upon introduction of GBP. **b** Representative structure of the OS^mod^ containing a benzene sulfonamide affinity tag. **c** 66-component OS^mod^ library used for CUPRA screening

Here, we present the competitive universal proxy receptor assay (CUPRA), a method that employs a library of heterobifunctional compounds, consisting of oligosaccharides linked to a common affinity tag, and ESI-MS to simultaneously measure coupled binding equilibria involving the library, a universal proxy protein receptor (^Uni^P_proxy_), which binds all components of the library through the affinity tag, and target GBP (Fig. 1a). Notably, the relative abundances of the ^Uni^P_proxy_–heterobifunctional ligand complexes measured by ESI-MS reflect the concentrations of free heterobifunctional ligands in solution. Changes in the relative abundances of the ^Uni^P_proxy_ complexes upon introduction of a GBP to solution serve to identify ligand binding to the GBP. Importantly, direct detection of the target GBP is not required and, consequently, CUPRA can be applied to any GBP, regardless of size or heterogeneity. Analysis of the magnitude of the changes, using a binding model that takes into account all possible interactions between the library components and GBP and ^Uni^P_proxy_, provides the affinities of the GBP ligands that are detected.

## Results

### Production of CUPRA ligand library

We introduced the CUPRA linker, which consists of an affinity tag based on the sulfonamide group and a short PEG dipeptide linker, to the reducing end of free oligosaccharides with an *N*-glycosidic linkage using established chemistry (Fig. 1b and Supplementary Fig. 1). The design of the linker and conjugation chemistry was dictated primarily by expediency of utilizing commercially available free oligosaccharides and a desire to preserve the pyranose form of reducing end monosaccharide. This library of modified oligosaccharides (OS^mod^) consists of 66 components; 62 contain oligosaccharide structures found in humans, including ganglio-, globo- and Lewis-oligosaccharides, blood group ABO antigens and human milk oligosaccharides (HMOs), as well as four plant oligosaccharides (Fig. 1c and Supplementary Table 1). The current library, although modest in size, contains the majority of commercially available human oligosaccharide determinants. We selected human carbonic anhydrase type 1, a 29 kDa monomeric metalloenzyme that binds the OS^mod^ with relatively high affinity at neutral pH (average *K*_d_ 13 ± 6 μM), as the ^Uni^P_proxy_ (Supplementary Table 2)^8^.

### Assay implementation and validation

The B subunit homotetramer of cholera toxin (CTB_5_) produced by *Vibrio cholera*, which binds with high affinity to the GM1 ganglioside^9^, the *Sambucus nigra* lectin (SNA), which is specific for α2-6-linked sialosides^10^, a fragment of family 51 carbohydrate binding module (CBM51), which recognizes A and B type 2 and 6 blood group antigens^11^, and a soluble form of human siglec-2 (CD22), which binds to α2-6 sialosides^12,13^, served as positive controls to validate CUPRA. We also investigated the *Maackia amurensis* lectins (MAA), a mixture of leucoagglutinin (MAL) and hemagglutinin (MAH). The preferred binding motif of MAH is reported to be Neu5Acα2-3Galβ1-3GalNAc (Neu5Ac ≡ 5–*N*-acetylneuraminic acid, Gal ≡ galactose, GalNAc ≡ galactosamine), while MAL preferentially binds structures with a Neu5Acα2-3Galβ1-4GlcNAc motif^14^. MAL tolerates substitution at C8 in Neu5Acα2-3 residue with another neuraminic acid residue, and both MAL and MAH can bind in a sialic acid-independent manner if sulfate is present on the underlying glycan^14^. The streptavidin homotetramer (S_4_), which does not bind glycans, served as a negative control.

**Fig. 2:**
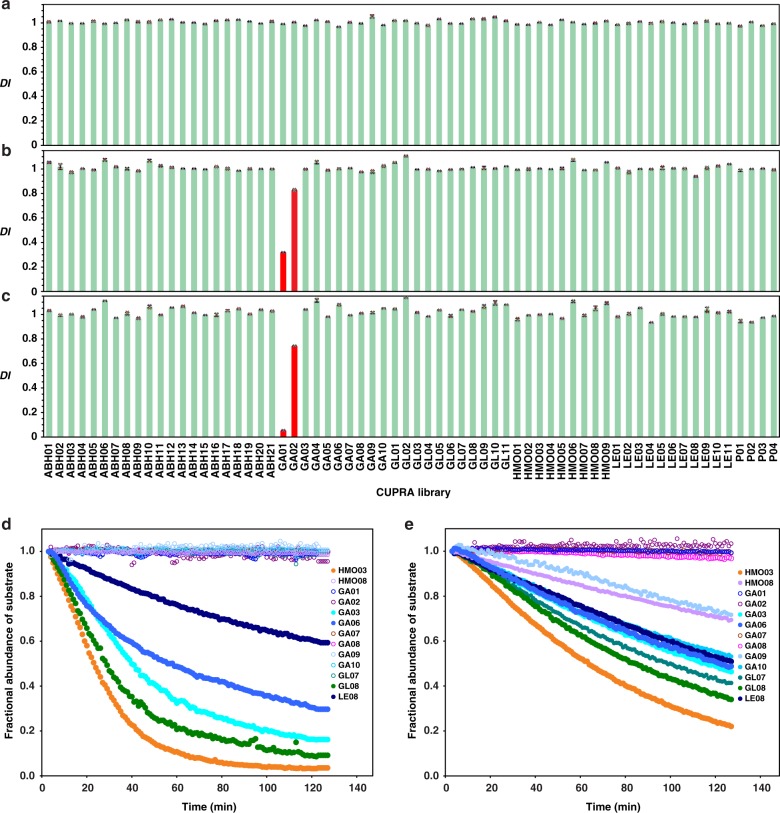
CUPRA enables screening of glycan libraries against GBPs and quantifying substrate specificity of CAZymes. **a** Library screening results for the negative control, streptavidin (50 μM). The standard deviation in individual *DI* values determined from four measurements is <0.03. **b**, **c** Glycan library screening against the positive control cholera toxin B subunit homopentamer (CTB_5_) at initial concentrations of 2 μM and 10 μM, respectively. Depleted library components are shown by red bars. **d**, **e** Time-dependent substrate fractional abundance measured by CUPRA for 13 Neu5Ac-containing OS^mod^ in the presence of human neuraminidase NEU2 and NEU3, respectively, at pH 7 and 25 °C. Error bars represent standard deviations calculated for *n* = 4 independent experiments

To implement CUPRA we performed ESI-MS on aqueous ammonium acetate (200 mM, pH 7) solutions of ^Uni^P_proxy_ (5 μM) and library (3 μM each) in the absence and presence of GBP (typically 1–50 μM). Unless otherwise noted, the measurements were performed at 25 °C. Ammonium acetate is the preferred buffer for protein–ligand binding measurements by ESI-MS and has been shown to be suitable for studying a wide variety of GBPs and their interactions^6,15–17^. The solution volume used for each analysis was ~3 μL; total acquisition time was ~2 min. We identified specific ligands from the depletion index (*DI*_*i*_, Eq. 1) – the change in the fractional abundance (*F*_*i*_, Eq. 2) ratio of ligand *i*-bound and free ^Uni^P_proxy_ ions upon addition of GBP. Prior to calculating *F*_*i*_, we treated each mass spectrum with the Sliding Window Adducts Removal Method (SWARM)^18^ to remove contributions to the abundance of ^Uni^P_proxy_ -OS^mod^ complex ions from adducts, formed by the ESI process, of neighbouring complex ions (Supplementary Fig. 2). Shown in Fig. 2a is a summary of the *DI*_*i*_ measured by CUPRA screening of the 66-component library against S_4_ (50 μM). As expected, the average *DI*_*i*_ for this negative control is close to 1.0 (0.99 ± 0.03). In contrast, CUPRA screening against the positive controls produced decreases in *DI*_*i*_ for one or more of the library components. CUPRA screening of the library against CTB_5_ (10 μM) identified the structurally-related **GA01** (GM1) and **GA02** (fucosyl-GM1) as ligands (Figs. 2b, c), while **ABH11**, **ABH12**, **ABH13**, **ABH15**, **ABH19** and **ABH20**, which contain A and B type 2 tetra- and pentasaccharides, were identified as ligands of CBM51 (Supplementary Fig. 3a). **HMO08** (composed of 6′-sialyllactose), the only compound in the library with α2-6-linked Neu5Ac, was identified as a ligand of SNA (Supplementary Fig. 3b). Application of CUPRA to human CD22, part of an important class of Neu5Ac-binding GBPs implicated in both innate and adaptive immunity^19^, correctly identified **HMO08** as a ligand (Supplementary Fig. 4a). CUPRA screening of MAA at high concentration (50 μM) identified **HMO03** and **GA06** as ligands. Both of these ligands contain the Neu5Acα2-3Galβ1-4Glc structure, although, in the case of **GA06**, it is capped by α2-8-linked Neu5Ac. The absence of detectable binding of MAA to **GA03**, which contains Neu5Acα2-3Galβ1-3GlcNAc, the preferred binding motif of MAH, suggests a much lower affinity for this monovalent interaction.

Affinities measured by CUPRA are summarized in Supplementary Table 3. Notably, the *K*_d_ for **GA01** (0.66 ± 0.01 μM), **GA02** (45 ± 4) binding to CTB_5_, for **ABH11** (96 ± 10 μM), **ABH12** (110 ± 10 μM), **ABH13** (170 ± 40 μM), **ABH15** (100 ± 10 μM), **ABH19** (97 ± 18 μM), and **ABH20** (92 ± 2 μM) binding to CBM51 and **HMO08** binding to CD22 (110 ± 20 μM) agree, within a factor of 6, with values determined by ESI-MS or ITC for the corresponding oligosaccharides^11–13,20^. The affinities of **HMO08** (0.43 ± 0.01 μM) for SNA and **HMO03** (9 ± 4 μM) and **GA06** (8.3 ± 3.0 μM) for MAA could not be confirmed by direct ESI-MS affinity measurements because of the heterogeneity of the GBPs; affinities measured by other in-solution assays have not been reported.

### Detecting low affinity interactions

The *K*_d_ for many monovalent GBP-glycan interactions is ~1 mM^21^. Such low affinity interactions are frequently missed in glycan array screening^22^ but can be detected with CUPRA performed at a high GBP concentration (in the range of 50 μM to 100 μM). However, such high concentrations may lead to GBP aggregation and precipitation. Implementation of variable-temperature CUPRA (Supplementary Fig. 5), at temperatures <25 °C also facilitates the detection of low affinity ligands owing to the non-negligible exothermicity of GBP-glycan interactions^23,24^. For example, at 0 °C we identified four additional ligands of CTB_5_ (**ABH9**, **GA09**, **LE09**, and **LE10**) and confirmed binding by direct ESI-MS measurements (Supplementary Fig. 6a). Notably, a survey of available glycan array data revealed that, of these four low affinity interactions, only that with the GD1b structure (found in **GA09**) was detected (Supplementary Fig. 6b). In the case of CD22, binding to **HMO08** was noticeably enhanced by decreasing the temperature to 0 °C (Supplementary Fig. 4).

### Screening of natural glycan libraries

An important feature of CUPRA is that it can be applied to natural glycan libraries, thereby greatly expanding the repertoire of glycan structures available for screening. To demonstrate this, we introduced CUPRA linker to a mixture of HMOs extracted from pooled donor milk. Before conjugation, HMOs with nine different MWs were identified (Supplementary Table 4 and Supplementary Fig. 7a) and the following compositions: Hex2Fuc (488.18 Da), Hex2Neu5Ac (633.22 Da), Hex3HexNAc (707.26 Da), Hex3HexNAcFuc (853.32 Da), Hex3HexNAcNeu5Ac (998.35 Da), Hex3HexNAcFuc2 (999.37 Da), Hex4HexNAc2Fuc (1218.45 Da), Hex4HexNAc2Fuc2 (1364.52 Da), Hex4HexNAc2Fuc3 (1510.56 Da) (Hex ≡ hexose, HexNAc ≡ *N*-acetylhexosamine, Fuc ≡ fucose). Attachment of CUPRA linker to components of each of the 9 HMO isomer sets was confirmed by ESI-MS (Supplementary Table 4 and Supplementary Fig. 7b). We then used CUPRA to screen this library against a C-terminal fragment of human galectin 3 (hGal-3C), for which the HMO binding specificities have been previously established^6^. CUPRA screening identified HMO ligands at four different MWs (corresponding to compositions Hex3HexNAc, Hex3HexNAcFuc, Hex4HexNAc2Fuc, Hex4HexNAc2Fuc2). Binding of HMOs from one or both of the isomer sets with compositions Hex3HexNAcNeu5Ac and Hex3HexNAcFuc2 was also established (Supplementary Fig. 8). However, because the MWs of these HMOs differ by only 1 Da, it was not possible to discriminate between these isomer sets when bound (as CUPRA ligands) to hGal-3C. Therefore, we treated them as a single isomer set.

Because the affinities of the CUPRA linker -modified HMOs (**HMO01** – **HMO09**) for ^Uni^P_proxy_ are similar (Supplementary Table 2), the total concentration of each HMO isomer set can be estimated from the relative abundances of the corresponding ^Uni^P_proxy_ complexes and, from this, the apparent affinity for hGal-3C calculated (Supplementary Table 5). Using an affinity of 7 μM for ^Uni^P_proxy_ (the average *K*_d_ for all HMOs in the CUPRA library), we found the apparent *K*_d_ for each of the isomer sets containing ligands are in the range of 3–87 μM. These findings are consistent with the results of HMO screening, which have shown the presence of high affinity (*K*_d_ < 10 μM) ligands corresponding to each of these isomer sets^4^.

### Quantifying substrate specificities of CAZymes

Finally, we demonstrated that CUPRA, when implemented in a time-resolved fashion, represents a straightforward method to establish substrate specificity of CAZymes. To apply CUPRA in this capacity, two or more substrates of interest are introduced, as their corresponding OS^mod^, to a solution containing the desired CAZyme. Changes in substrate concentrations, as well as those of the corresponding enzyme products, are determined from the relative abundances of OS^mod^-bound ^Uni^P_proxy_ measured by time-resolved ESI-MS. Because of the manner in which the substrate and product concentrations are measured, the assay is insensitive to differences in their ESI-MS response factors and, consequently, independent of the nature of the chemical modification catalyzed by the enzyme. As a result, time-resolved CUPRA eliminates the need for calibration curves or internal standards, which are generally required with ESI-MS-based enzyme kinetics assays^25^.

Two glycosyl hydrolases, human neuraminidase 2 (NEU2) and 3 (NEU3), which preferentially cleave α2-3-linked Neu5Ac residues^26^, were used to illustrate the ease with which time-resolved CUPRA can establish substrate specificities. We incubated libraries of the Neu5Ac-containing OS^mod^ with NEU2 (Fig. 2d) or NEU3 (Fig. 2e) and monitored their conversion to the corresponding desialylated OS^mod^ products at pH 7 and 25 °C. Under these conditions, the time-resolved CUPRA data clearly show that both NEU2 and NEU3 exhibit a preference for **HMO03** (Supplementary Table 6). All OS^mod^ containing terminal α2-3-linked Neu5Ac were substrates of NEU3, with initial rates that are 10–50% that of **HMO03**. Those with α2-6-linked Neu5Ac were worse substrates for both enzymes (e.g. **HMO08**), consistent with previous reports^26^. Interestingly, all α2-8-linked Neu5Ac residues were substrates for NEU3 (**GA06**, **GA09**, **GA10**), but only **GA06** was a substrate for NEU2. Substrates containing branching after the α2-3-linkage were poor substrates for both enzymes. NEU2 did not tolerate Neu5Acα2-3 linked to Galα1-4Gal in **GL07**. Substrates with internal Fuc residues (e.g. **LE08**) were better tolerated by NEU3, compared to NEU2, in contrast to reported results for a structurally-similar substrate (Neu5Acα2-3Galβ1-4GlcNAc(Fucα2-3))^26^. We note, however, that previous reports have examined NEU2 and NEU3 activity under more acidic solution conditions. Furthermore, the nature of the substrate aglycone may influence the activity of human neuraminidases^25^.

## Discussion

The absence of a high-throughput and quantitative method for glycan library screening substantially impedes glycomics research as screening results must currently be followed by time- and sample-intensive affinity measurements. In this work, we introduced CUPRA, a new screening method that combines direct ESI-MS binding measurements and a library of novel heterobifunctional compounds (oligosaccharides with a common affinity tag), which overcomes this obstacle. Application of CUPRA to screen a library of 66 glycan structures against a series of human, plant and bacterial GBPs glycan-binding proteins, demonstrated the ability of assay to rapidly identify and quantify ligands. Importantly, when implemented at low temperature (0 °C), CUPRA readily allows for detection of low affinity (*K*_d_ ≤ 1 mM) interactions. Notably, the assay can be applied to both defined and natural glycan libraries. The use of natural libraries substantially enhances the diversity of glycan structures available for screening and, thereby, facilitates the identification of the natural ligands of GBPs.

The ability to implement CUPRA in a time-resolved manner yields a remarkably simple method to measure the activities of CAZymes. Because the assay can be used to monitor enzymatic conversion of multiple substrates simultaneously (in the same solution), substrate specificity of CAZymes can be quantified with a precision not generally accessible with existing kinetic assays. The tremendous potential of CUPRA was demonstrated for two glycosyl hydrolases, the human sialidases NEU2 and NEU3. However, the approach can be readily extended to other classes of CAZymes.

In summary, CUPRA represents a powerful and exceptionally versatile addition to the glycomics researcher toolbox, one that is expected to dramatically accelerate the discovery and quantification of glycan-GBP interactions and characterization of CAZymes.

## Methods

### Proteins

Cholera toxin B subunit homopentamer from *Vibrio cholerae* (CTB_5_, 58,020 Da, purity > 95%), *Maackia amurensis* agglutinin (MAA, 130 kDa, purity > 85%) and *Sambucus nigra* (SNA, 140 kDa, purity > 90%) lectins were purchased from Sigma-Aldrich (Canada). A gene fragment encoding a family 51 carbohydrate-binding module (CBM51, MW 20 735 Da, purity > 95%) was recombinantly produced in *Escherichia coli* and purified as described elsewhere^27^. Residues 1–332 of human Siglec-2 (MW 140 kDa, purity > 95%) were cloned in frame with human IgG1 Fc and a C-terminal His_6_, as described previously^28^. This chimeric construct, in the *pcDNA5/FRT* vector, was stably transfected into *Chinese Hamster Ovary* Lec-1 cell line through *Flp-In* system under selection with 0.5 mg mL^−1^ hygromycin-B for ~2 weeks. For expression, cells were grown in T-175 flasks for 12 d after reaching confluency, in 50 mL of DMEM-F12 media containing 10% FBS, 0.5% penicillin-streptomycin, and 1% HEPES. The protein supernatant was harvested, centrifuged (300 rcf, 10 min) and sterilized through a 0.5 μM filter for storage at 4 °C. For purification, 130 mL of the supernatant was loaded at 1 mL mL-1 onto a 1 mL Histrap Excel column (GE healthcare) equilibrated with 20 mM sodium phosphate, 0.5 M NaCl at pH 7.4. After loading, the column was washed with 15 mL of 30 mM imidazole in 20 mM sodium phosphate, 0.5 M NaCl at pH 7.4, then eluted with 500 mM imidazole in 20 mM sodium phosphate, 0.5 M NaCl, at pH 7.4. Fractions containing protein were diluted 10-fold in 20 mM phosphate buffer at pH 7.0. The diluted fractions were loaded onto a Protein-G column (GE healthcare) equilibrated with 20 mM phosphate buffer. The loaded protein on the column was washed with 15 mL of 20 mM phosphate buffer (pH 7.0) and eluted with 100 mM glycine solution at pH 2.7 via syringe and neutralized with 40-50 µL of 1 M Tris buffer at pH 9.0 per 1 mL fraction. Fractions containing protein were dialyzed into 2 L of 200 mM ammonium acetate three times. Finally, the protein was concentrated, by centrifuging (19,000 rcf) through a 30 kDa molecular weight cutoff filter, to approximately 3.5 mg mL^−1^ in 50 µL. The galectin-3 carbohydrate recognition domain (hGal-3C; amino acid residues 107–250) was expressed in *E. coli* BL21(DE3) as previously described^29^. Human neuraminidase enzymes NEU2 and NEU3 were expressed as fusion proteins with maltose-binding protein and purified as previously described^25^. Enzyme activity was determined in comparison to a standard curve of neuraminidase from *Clostridium perfringens* against the fluorogenic substrate 4-methylumbelliferyl α-D-*N*-acetylneuraminic acid^30^. Fluorescence was measured on a Spec-traMax M2e plate reader (Molecular Devices), excitation 357 nm and emission 434 nm.

### Oligosaccharides

Free reducing oligosaccharides (Supplementary Table 1) corresponding to the structures found in **ABH02** – **ABH21**, **GA01**-**GA10**, **GL01** – **GL11**, **HMO02** – **HMO07**, **HMO09** and **LE01** – **LE11** were purchased from Elicityl SA (Crolles, France); the oligosaccharide used for ABH02 was purchased from Dextra (Reading, UK); those for **HMO01** and **P01** – **P04** were purchased from Sigma-Aldrich Canada (Oakville, ON, Canada) and the oligosaccharide used to produce HMO08 was purchased from Carbosynth (San Diego, CA, USA). HMO fractions were prepared and purified as described elsewhere^31^. Briefly, pooled (four donors) human milk (1 L) was centrifuged at 5000 RCF for 30 min at 4 °C, and the fat was removed. Ethanol (2 L) was added and the solution was incubated overnight at 24 °C. The precipitate was removed by centrifugation at 5000 RCF for 30 min at 4 °C, and the solvent was removed by rotary evaporation. The HMO fraction was dissolved in 5 mL of water and the solution was passed through a Bio Gel P-2 (Extrafine, < 45 μm; Bio-Rad Laboratories, Hercules, CA) column (2.6 × 100 cm). Elution was performed with 100 mM aqueous ammonium acetate at a flow rate of 26 mL h^−1^, and the elution profile was recorded with a refractive index detector (Waters, differential refractometer R401). A total of six (I–VI) HMO fractions were collected and freeze dried. Fractions III and IV were used in the current work.

### Synthesis of CUPRA linker and preparation of OS^mod^ library

The CUPRA linker was designed with an affinity tag based on the sulfonamide group and a short PEG dipeptide linker to facilitate purification of the resulting OS^mod^ (Supplementary Fig. 1). Two phenylalanine residues were incorporated to ensure sufficient retention on reverse phase media. Solid phase-assisted assembly of the linker flanked by a sulfonamide moiety and a free amine was done on trityl chloride polystyrene resin. Each incubation and washing step was performed in a glass column under a flow of N_2_ supplied through the glass frit on the bottom. Each washing step was performed 3 times with DMF, followed by vacuum aspiration through the glass frit. A 2,2′-(ethanedioxy)bis(ethylamine) (3 eq.) solution in DMF was added to the resin and incubated for 1 h followed by washing. Fmoc-phenylalanine (3 eq.), HBTU (3 eq.) and DIPEA (3.3 eq.) were added and incubated with the resin for 30 min, followed by washing with DMF. Fmoc deprotection was performed by incubating the resin with a solution of 20% piperidine in DMF for 20 min, followed by washing with DMF. Coupling with Fmoc-phenylalanine and deprotection was repeated as described above. Glutaric anhydride (3 eq.) was added and incubated for 1 h, followed by washing with DMF. 4-(2-aminoethyl)benzenesulfonamide (3 eq.), HBTU (3 eq.) and DIPEA (3.3 eq.) were added, incubated for 1 h, then washed. After washing the resin 3 times with DCM the product (linker) was cleaved using 50% TFA and concentrated using a rotary evaporator. Purification on C-18 HPLC column in gradient of water (0.1% TFA) – MeCN (0.1% TFA) and concentration gave residue, which was taken up into water-MeOH (1:1), treated with Dowex (HCO_3_− form) to convert to free amine, concentrated and freeze dried.

A two step, one-pot procedure, was used to generate library components. Amination of the anomeric center of the reducing sugars was performed using a primary amine followed by acetylation of the resulting *N*-glycoside. A sample of oligosaccharide (2–4 mg) was placed in 0.6 mL Eppendorf vial. The CUPRA linker (10 μL, 0.5 M in DMSO) was added, the vial was vortexed then incubated at 50 °C for 16–24 h. The mixture was diluted with DMSO (80 μL), acetic anhydride  (80 μL) was then added and the mixture was incubated for 3–4 h, then diluted with water and purified on a C-18 HPLC column in a gradient of water (0.1% TFA) – MeCN (0.1% TFA); appropriate fractions were concentrated and freeze dried.

### Mass spectrometry

Synapt G2 and a G2S quadrupole-ion mobility separation-time-of-flight (Q-IMS-TOF) mass spectrometers (Waters UK Ltd., Manchester, UK), each equipped with a nanoflow ESI (nanoESI) source, were used^32^. All measurements were carried out in positive ion mode. To perform nanoESI, 5 μL of solution was loaded into a nanoESI tip (~5 μm o.d.), which was produced in-house from a borosilicate capillary (1.0 mm o.d., 0.68 mm i.d.) using a P-1000 micropipette puller (Sutter Instruments, Novato, CA). To initiate the spray, a voltage of ~1.0 kV was applied to a platinum wire inserted into the nanoESI tip. Cone, Trap and Transfer voltages of 20, 3, and 1 V, respectively, were used^32^. External mass calibration was carried out using an aqueous CsI (1 mg mL^−1^) solution. Mass spectra (consisting of at least 150 scans) were acquired and processed using MassLynx (v 4.1). The temperature of the solution in the nanoESI tip was controlled using a home-built device (Supplementary Fig. 5). Cooling of the nanoESI tip, which was inserted into a central channel in the device, was achieved by passing cooled nitrogen gas through the two symmetric gas flow channels in the aluminum block. The temperature of the solution was determined from a thermocouple placed in proximity to the end of the nanoESI tip.

Prior to calculating *F*_*i*_, each mass spectrum was treated with SWARM^18^ to remove contributions to the abundance of ^Uni^P_proxy_ – ligand complex ions from adducts, formed by the ESI process, of neighboring complex ions. This spectral ‘cleaning’ procedure facilitates the detection of small changes in ion abundances associated with low affinity interactions. Briefly, each spectrum was smoothed using Savitzky-Golay filter of order 4 and window 41. After enumeration and identification of peaks corresponding to ^Uni^P_proxy_ – OS^mod^ complexes, the portion of the mass spectrum corresponding to the adducts associated with the ^Uni^P_proxy_–OS^mod^ complex with the smallest *m/z* was subtracted from the original mass spectrum. The adduct distribution was then subtracted, in a stepwise fashion from all ^Uni^P_proxy_–OS^mod^ complexes, in order of increasing *m/z*. In each step, the maximum abundance of the distribution (corresponding to the ^Uni^P_proxy_ – OS^mod^ complex free of adducts) was rescaled to match that of the other complexes. This procedure was performed in a charge state dependent fashion. In some cases, background was subtracted before and after SWARM using an asymmetric least square smoothing method^33^.

Using SWARM-treated mass spectra, *DI*_*i*_ was calculated for each ligand from the ratio of the fractional abundances (*F*_*i*_) of ligand (*i*)-bound and free ^Uni^P_proxy_ ions in the presence (+GBP) and absence (-GBP) of GBP, Eqs. (1) and (2):

1 $$DI_i = \frac{{F_i( + {\mathrm{GBP}})}}{{F_i( - {\mathrm{GBP}})}}$$

2 $$F_i = \frac{{Ab({\,}^{{\mathrm{Uni}}}{\mathrm{P}}_{{\mathrm{proxy}}}{\mathrm{L}}_i)}}{{\mathop {\sum}\limits_i {Ab({\,}^{{\mathrm{Uni}}}{\mathrm{P}}_{{\mathrm{proxy}}}{\mathrm{L}}_i)} }}$$

### ESI-MS affinities

The affinity (*K*_d,proxy,*i*_, Eq. 3) of each CUPRA library component (L_*i*_) for ^Uni^P_proxy_ was quantified using the direct ESI-MS assay^34^. The reported affinities are average values from six replicate measurements performed at a minimum of three different ^Uni^P_proxy_ and L_*i*_ concentrations. The reference protein method was used to correct, when needed, the mass spectra for the  occurrence of nonspecific binding of L_*i*_ to ^Uni^P_proxy_ during the ESI process^35^. *K*_d,proxy,*i*_ was calculated from the total abundance (*Ab*) ratio (*R*_*proxy,i*_, Eq. 4) of the ligand-bound (^Uni^P_proxy_L_*i*_)-to-free protein (^Uni^P_proxy_) ions and the initial concentrations of ^Uni^P_proxy_ ([^Uni^P_proxy_]_0_) and ligand ([L_*i*_]_0_).

3 $${\it{K}}_{{\mathrm{d},{{\mathrm{proxy}},}}i} = \frac{{[{\mathrm{L}}_i]_0}}{{R_{{\mathrm{proxy}},i}}} - \frac{1}{{{\mathrm{1}} + R_{{\mathrm{proxy}},i}}}\left[ {{\,}^{{\mathrm{Uni}}}{\mathrm{P}}_{{\mathrm{proxy}}}} \right]_0$$

4 $$R_{{\mathrm{proxy}},i} = \frac{{Ab\left( {{\,}^{{\mathrm{Uni}}}{\mathrm{P}}_{{\mathrm{proxy}}}{\mathrm{L}}_i} \right)}}{{Ab\left( {{\,}^{{\mathrm{Uni}}}{\mathrm{P}}_{{\mathrm{proxy}}}} \right)}} = \frac{{\left[ {{\,}^{{\mathrm{Uni}}}{\mathrm{P}}_{{\mathrm{proxy}}}{\mathrm{L}}_i} \right]}}{{\left[ {{\,}^{{\mathrm{Uni}}}{\mathrm{P}}_{{\mathrm{proxy}}}} \right]}}$$

The affinity of a given L_*i*_ for a GBP was determined in the same way. For CTB_5_, which has multiple binding sites, the procedure was adapted as described previously^20^.

### CUPRA affinities

To implement CUPRA we performed ESI-MS measurements on aqueous ammonium acetate solutions (pH 7, 25 °C) of ^Uni^P_proxy_ (5 μM) and library (typically 8–10 components, 3 μM each) in the absence and presence of GBP (typically 1 μM to 50 μM). The solution volume used for each analysis was ~3 μL; total acquisition time was ~2 min. The affinity of a given L_*i*_ for a target GBP (*K*_d,GBP,*i*_, Eq. 5) was calculated from the measured *R*_proxy_,_*i*_ and mass balance considerations, Eqs. 6–11.

5 $${\it{K}}_{{\mathrm{d,GBP}},i} = \frac{{[{\mathrm{GBP}}][{\mathrm{L}}_i]}}{{[{\mathrm{GBPL}}_i]}}$$

6 $$\left[ {{\,}^{{\mathrm{Uni}}}{\mathrm{P}}_{{\mathrm{proxy}}}} \right]_0 = \left[ {{\,}^{{\mathrm{Uni}}}{\mathrm{P}}_{{\mathrm{proxy}}}} \right] + \mathop {\sum}\limits_i {\left[{{\,}^{{\mathrm{Uni}}}{\mathrm{P}}_{{\mathrm{proxy}}}{\mathrm{L}}_i} \right]}$$

7 $$\left[ {{\,}^{{\mathrm{Uni}}}{\mathrm{P}}_{{\mathrm{proxy}}}} \right] = \frac{{\left[ {{\,}^{{\mathrm{Uni}}}{\mathrm{P}}_{{\mathrm{proxy}}}} \right]_0}}{{1 + \mathop {\sum}\limits_i {R_{{\mathrm{proxy}},i}} }}$$

8 $$\left[ {{\,}^{{\mathrm{Uni}}}{\mathrm{P}}_{{\mathrm{proxy}}}{\mathrm{L}}_i} \right] = \frac{{R_{{\mathrm{proxy}},i}\left[ {{\,}^{{\mathrm{Uni}}}{\mathrm{P}}_{{\mathrm{proxy}}}} \right]_0}}{{1 + \mathop {\sum}\limits_i {R_{{\mathrm{proxy}},i}} }}$$

9 $$[{\mathrm{L}}_i] = \frac{{{\it{K}}_{{\mathrm{d,proxy,}}i}}}{{R_{{\mathrm{proxy}},i}}}$$

10 $$\left[ {{\mathrm{GBPL}}_i} \right] = [{\mathrm{L}}_i]_0 - [{\mathrm{L}}_i] - \left[ {{\,}^{{\mathrm{Uni}}}{\mathrm{P}}_{{\mathrm{proxy}}}{\mathrm{L}}_i} \right]$$

11 $$[{\mathrm{GBP}}] = [{\mathrm{GBP}}]_0 - \mathop {\sum}\limits_i {[{\mathrm{GBPL}}_i]}$$

### Enzyme kinetics

The time-resolved CUPRA measurements were performed by manually mixing aliquots of stock solutions of NEU2 or NEU3 (final concentration 2 µM), ^Uni^P_proxy_ (5 µM), substrates (S_*i*_, 5 µM each) and CUPRA linker (5 µM), all in 200 mM aqueous ammonium acetate at pH 7 and 25 °C. ESI mass spectra were collected continuously at the rate of 30 scans min^−1^ starting at 3 min after mixing. The adduct distributions measured for the ^Uni^P_proxy_ – CUPRA linker complex ions were used to apply SWARM. The time-dependent fractional abundance of a given substrate (F_S,*i*_) (Eq. 12), which was used to represent reaction progress, was calculated from the total *Ab* of ^Uni^P_proxy_ bound to S_*i*_ and corresponding product (P_*i*_), corrected for S_*i*_ consumed prior to data acquisition.

12 $$F_{S,i} = \frac{{Ab\left( {{\,}^{{\mathrm{Uni}}}{\mathrm{P}}_{{\mathrm{proxy}}}{\mathrm{S}}_i} \right)}}{{Ab\left( {{\,}^{{\mathrm{Uni}}}{\mathrm{P}}_{{\mathrm{proxy}}}{\mathrm{S}}_i} \right) + Ab\left( {{\,}^{{\mathrm{Uni}}}{\mathrm{P}}_{{\mathrm{proxy}}}{\mathrm{P}}_i} \right)}}$$

### Statistical analysis

Statistical analyses of experimental data were conducted using two-tailed Student’s *t* test. A *p* value < 0.05 was accepted as statistically significant. Values are expressed as the mean ± standard deviation.

### Reporting summary

Further information on research design is available in the Nature Research Reporting Summary linked to this article.

## Supplementary information

Description of additional supplementary items

Supplementary Information

Reporting Summary

Supplementary Data 1

## Data Availability

The data that support the findings of this study are available from the corresponding author upon reasonable request. The source data underlying Fig. 2 and Supplementary Figs. 3–6 are shown in Supplementary Data 1.

## Code availability

Custom code used to generate the findings of the study is available at https://github.com/pkitov/CUPRA-SWARM.
