# Supplementary material for: A quantitative, high-throughput method identifies protein–glycan interactions via mass spectrometry
Source: Commun Biol. 2019 Jul 22;2:268. doi: 10.1038/s42003-019-0507-2 (PMC6646405; doi:10.1038/s42003-019-0507-2)
Supplement: Supplementary file 1 — Description of additional supplementary items [file 42003_2019_507_MOESM1_ESM.docx]

**Description of Additional Supplementary Files**

**File Name**: Supplementary Data 1

**Description**: The excel file contains the tabulated data (including standard deviations and associated p values) shown in Fig 2 and Supplementary Figures 3-6.
