# Supplementary material for: A quantitative, high-throughput method identifies protein–glycan interactions via mass spectrometry
Source: Commun Biol. 2019 Jul 22;2:268. doi: 10.1038/s42003-019-0507-2 (PMC6646405; doi:10.1038/s42003-019-0507-2)
Supplement: Supplementary file 2 — Supplementary Information [file 42003_2019_507_MOESM2_ESM.pdf]

Supplementary Figures

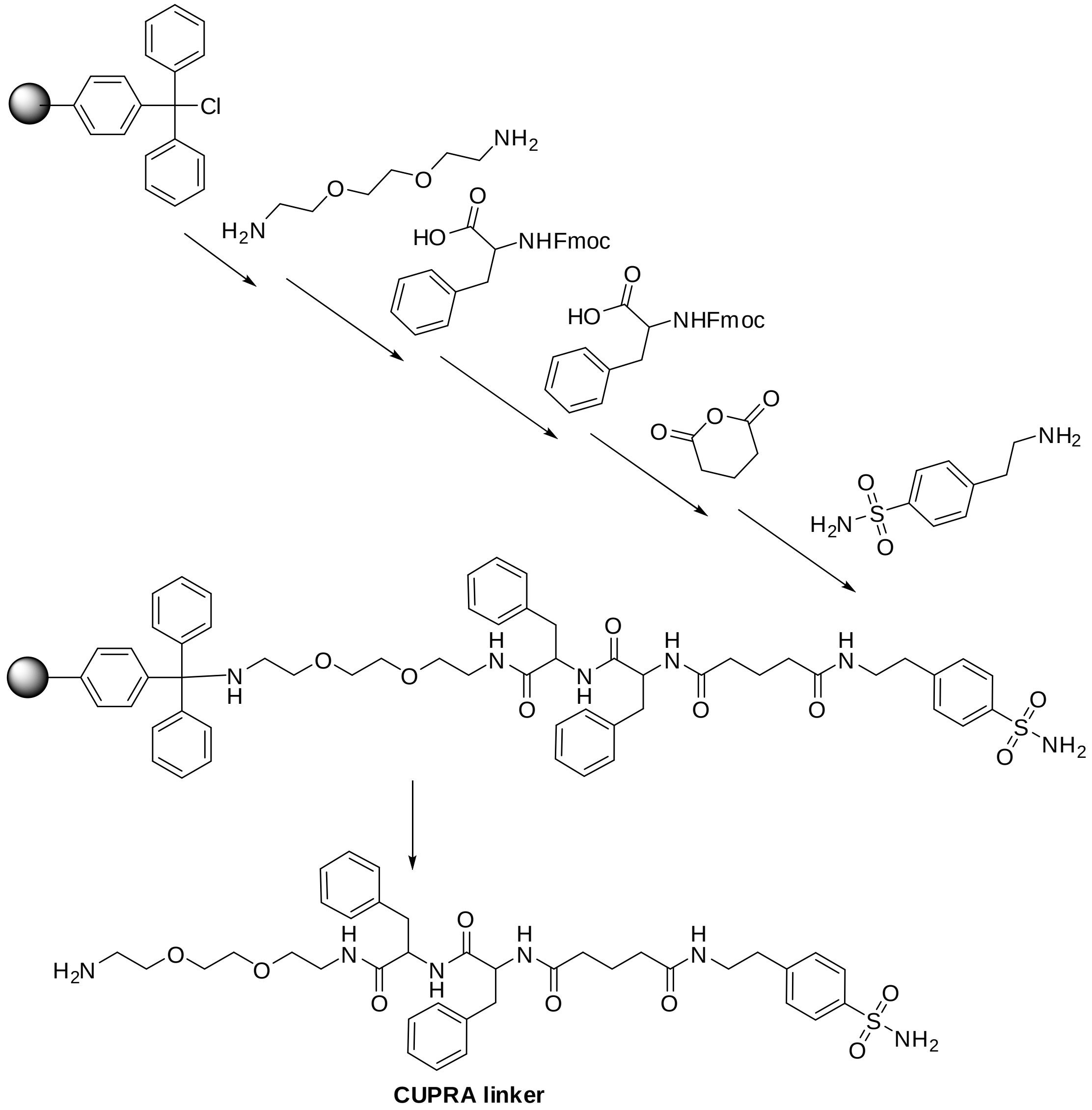

Supplementary Figure 1. Scheme of solid-phase assisted synthesis of CUPRA linker.

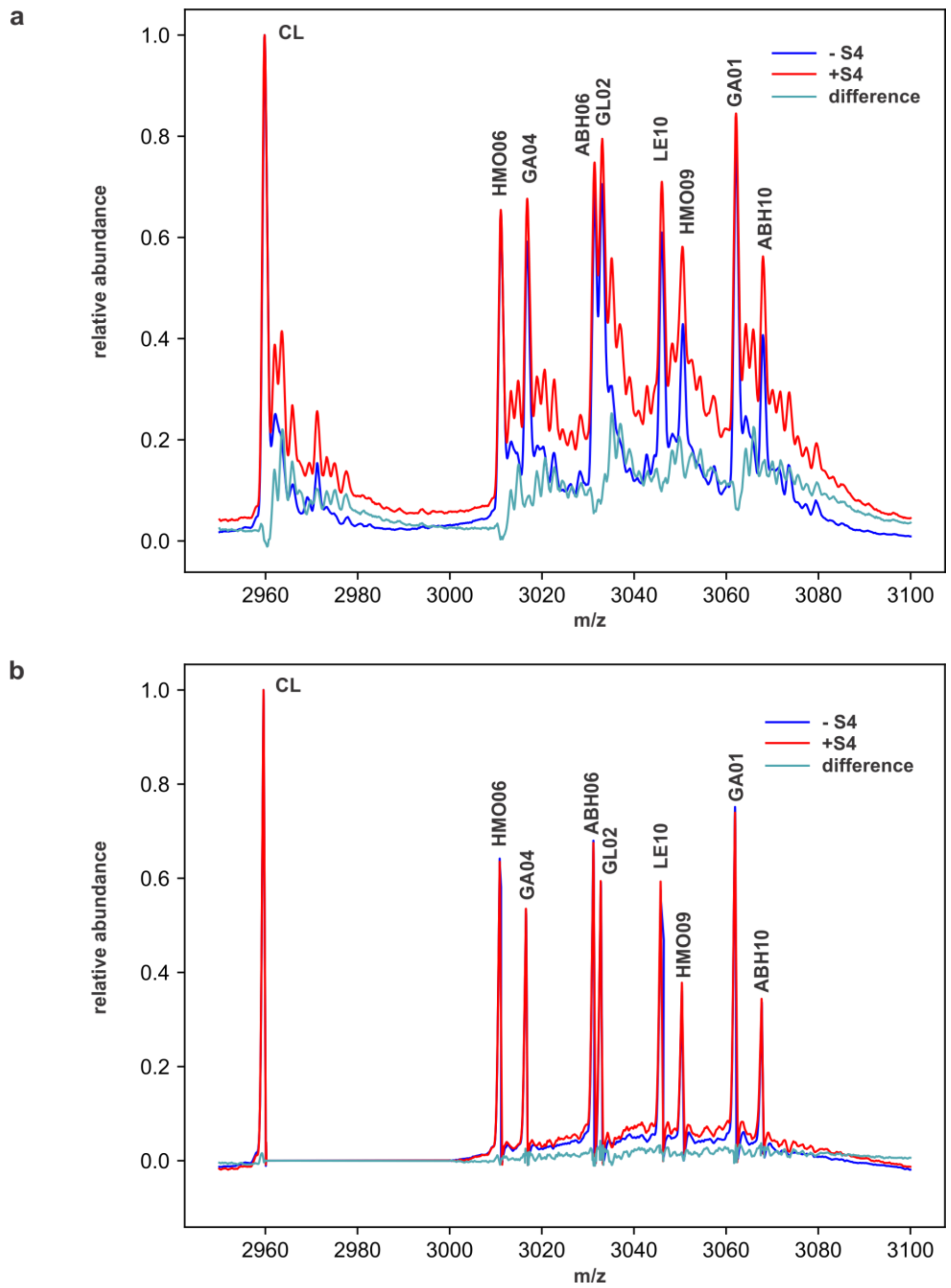

Supplementary Figure 2. Application of SWARM for adduct subtraction from ESI mass spectra. (a) Portion of ESI mass spectrum acquired for an aqueous ammonium acetate (200 mM, pH 7) solution of $^{Uni}P_{proxy}$ (3 μM), a subset of the OS^{mod} library (3 μM each) and CUPRA linker (**CL** 3 μM) in the absence (red trace) and presence (blue trace) of S_4 (50 μM). Cyan trace corresponds to difference between these two mass spectra. (b) Data from (a) after treatment with SWARM.

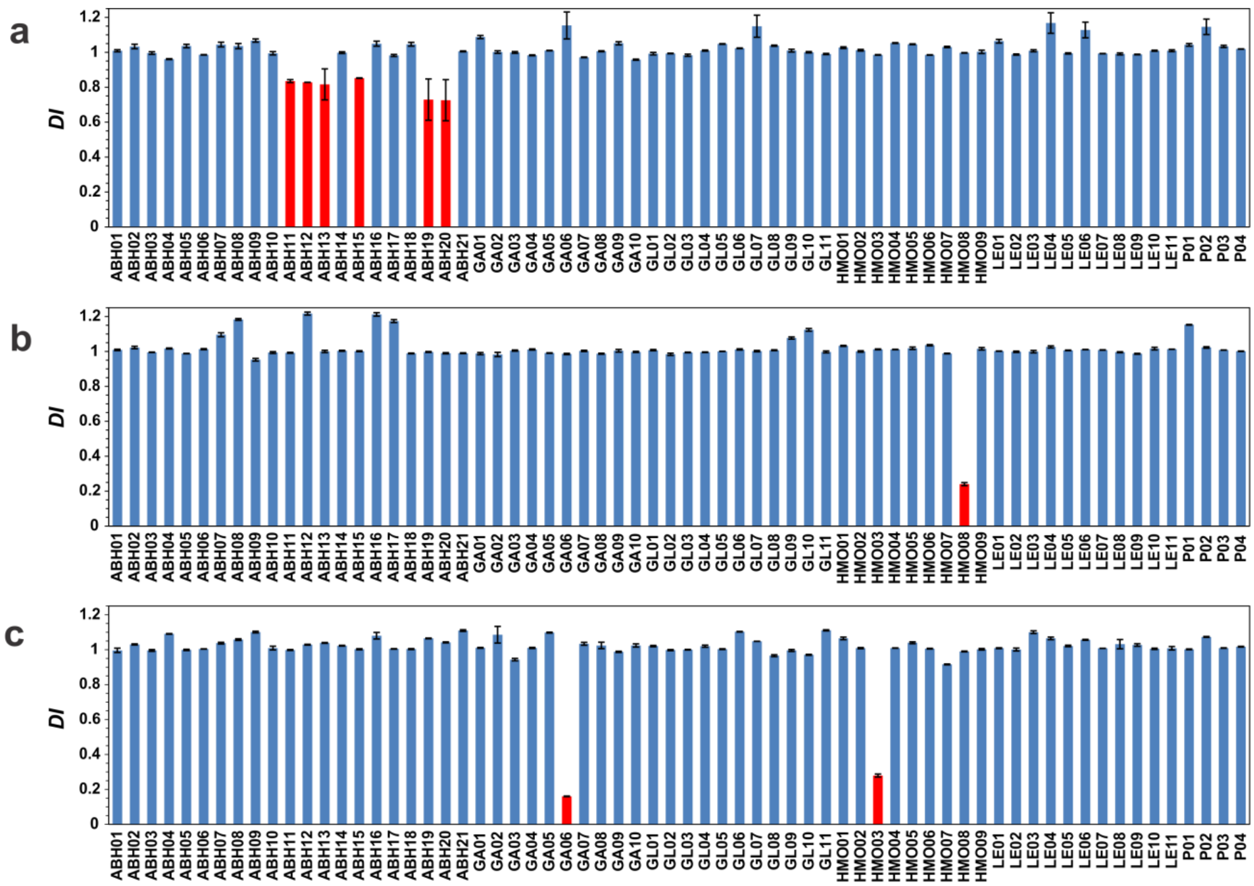

Supplementary Figure 3. CUPRA screening results for CBM51, SNA, and MAA. Depletion index (*DI*) for each OS^{mod} measured in 200 mM aqueous ammonium acetate (pH 7) for: (a) CBM51 (25 μM, 25 ºC), (b) SNA (5 μM, 25 ºC), (c) MAA (50 μM, 25 ºC). Data shown in red indicate ligands identified from screening. Error bars represent standard deviations calculated for n=4 independent experiments.

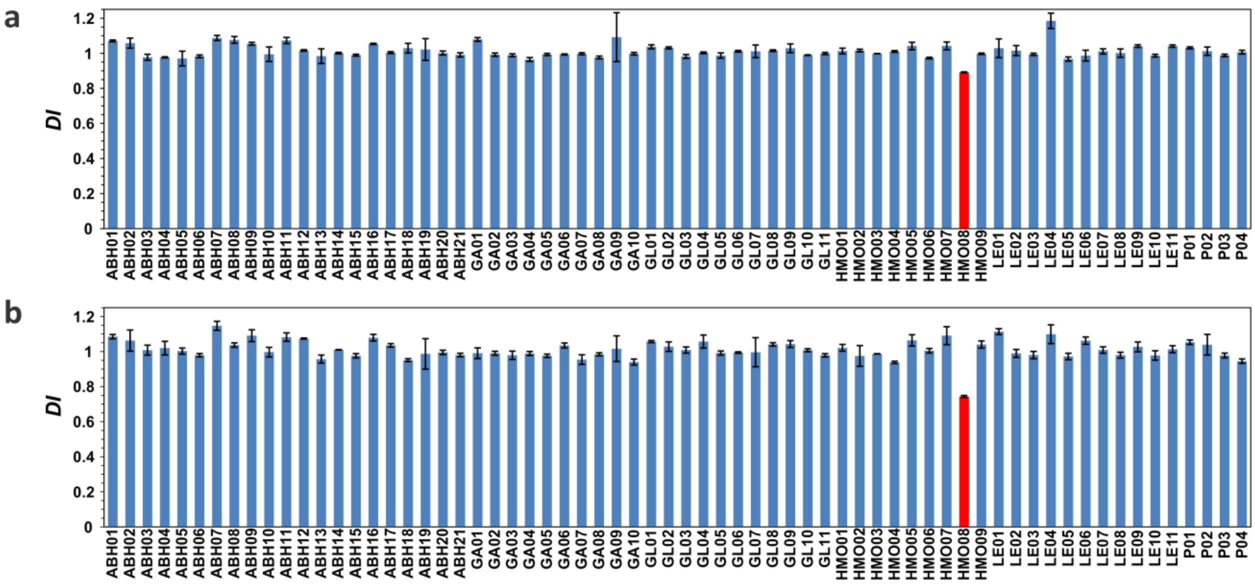

Supplementary Figure 4. CUPRA screening results for siglec-2. Depletion index (*DI*) for each OS^{mod} measured in 200 mM aqueous ammonium acetate (pH 7) for siglec-2 (23 μM) at: (a) 25 ^{o}C and (b) 0 ^{o}C. Data shown in red indicate ligands identified from screening. Error bars represent standard deviations calculated for n=4 independent experiments.

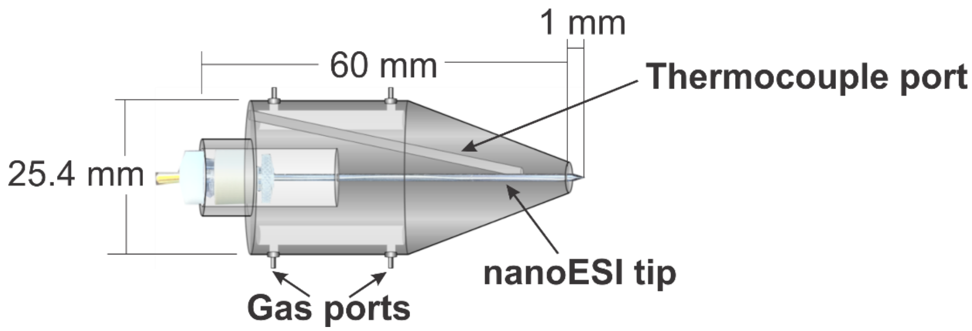

Supplementary Figure 5. Schematic of home-built temperature-controlled nanoESI device. The nanoESI tip is inserted into the central channel of the aluminum device through a small aperture 1.2 mm (i.d.). The tip is positioned such that ~1 mm of the tapered end protrudes from the end of the device. The temperature of the device was regulated by varying the flow rate of nitrogen gas that first passes through a U-tube in contact with a dry ice/ethanol bath and then through two symmetric gas flow channels at the outer edges of the device. The temperature of the device was measured by a thermocouple introduced through the angled channel and placed in proximity to the end of the nanoESI tip.

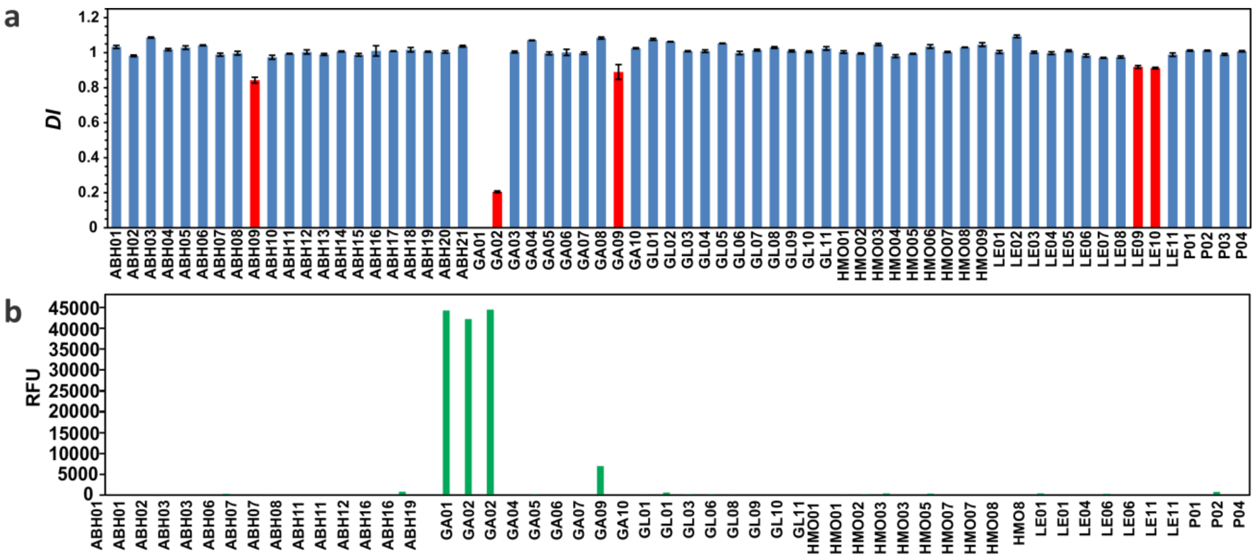

Supplementary Figure 6. Comparison of variable temperature CUPRA screening results with glycan array data for CTB_5: (a) Depletion index (*DI*) for each OS^{mod} measured in 200 mM aqueous ammonium acetate (pH 7) for CTB_5 (10 µM). Data shown in red indicate ligands identified from screening. Error bars represent standard deviations calculated for n=4 independent experiments. (b) Partial readout in relative fluorescence units (RFU) of CFG array (entry CTB_14501_10ug_v5.0_DATA.xls) screened against CTB_5 (only compounds containing glycans found in CUPRA library are shown). CUPRA codes instead of CFG codes are used for ease of comparison.

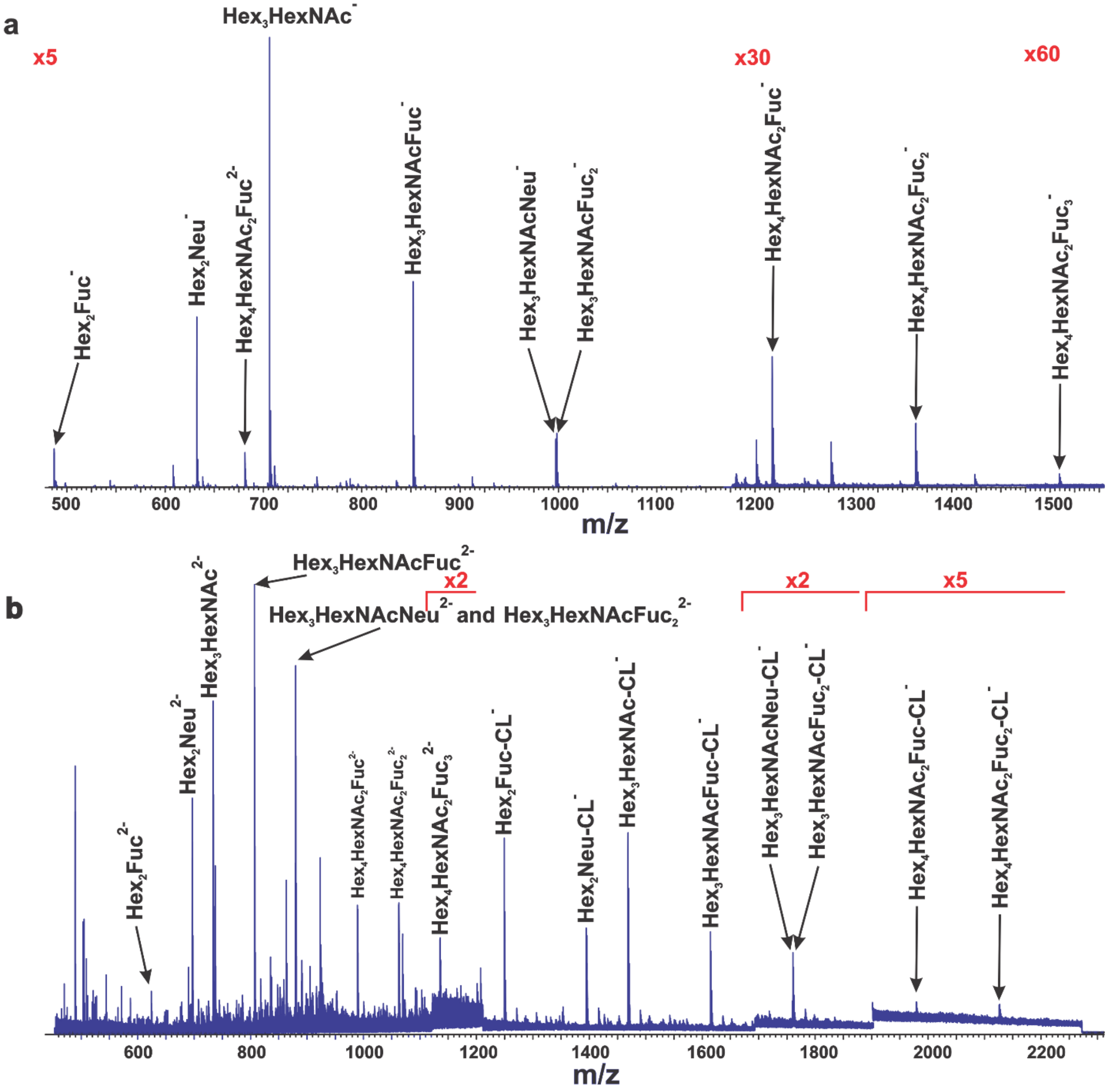

Supplementary Figure 7. ESI mass spectra of 200 mM aqueous ammonium acetate (pH 7) solution of 0.04 mg/ml HMO library before (a) and after (b) modification with CUPRA linker (**CL**).

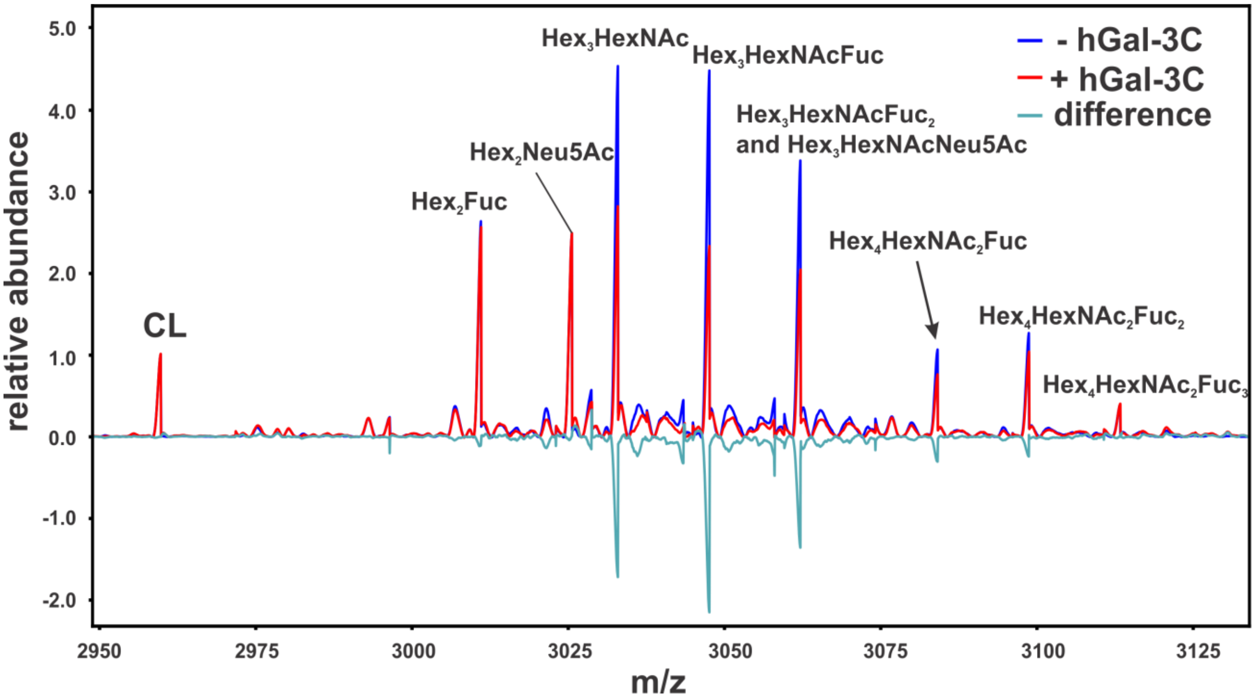

Supplementary Figure 8. Portion of ESI mass spectrum acquired for an aqueous ammonium acetate (200 mM, pH 7) solution of $^{Uni}P_{proxy}$ (3 μM), a subset of the HMO library modified with CUPRA linker (**CL**) (0.08 mg/ml) and CUPRA linker (**CL**, 3 μM) in the absence (red trace) and presence (blue trace) of hGal-3C (40 μM). Cyan trace corresponds to difference between these two mass spectra. All mass spectra were treated with SWARM.

Supplementary Tables

Supplementary Table 1. Structures of CUPRA library components (OS^{mod}); **CL** (CUPRA linker moiety) represents the linker-affinity tag.

OS^{mod}	**Structure**
**ABH01**	Galβ1-3GlcNAc-CL
**ABH02**	Galα1-3Galβ1-4GlcNAc-CL
**ABH03**	Fucα1-2Galβ1-3GlcNAcβ1-3Galβ1-4Glc-CL
**ABH04**	Fucα1-2Galβ1-4GlcNAcβ1-3Gal-CL
**ABH05**	Fucα1-2Galβ1-4GlcNAcβ1-3Galβ1-4Glc-CL
**ABH06**	Fucα1-2Galβ1-3GalNAcβ1-3Gal-CL
**ABH07**	GalNAcα1-3(Fucα1-2)Gal-CL
**ABH08**	GalNAcα1-3(Fucα1-2)Galβ1-3GlcNAc-CL
**ABH09**	GalNAcα1-3(Fucα1-2)Galβ1-3GlcNAcβ1-3Gal-CL
**ABH10**	GalNAcα1-3(Fucα1-2)Galβ1-3GlcNAcβ1-3Galβ1-4Glc-CL
**ABH11**	GalNAcα1-3(Fucα1-2)Galβ1-4GlcNAc-CL
**ABH12**	GalNAcα1-3(Fucα1-2)Galβ1-4GlcNAcβ1-3Gal-CL
**ABH13**	GalNAcα1-3(Fucα1-2)Galβ1-4GlcNAcβ1-3Galβ1-4Glc-CL
**ABH14**	GalNAcα1-3(Fucα1-2)Galβ1-3GalNAcβ1-3Gal-CL
**ABH15**	GalNAcα1-3(Fucα1-2)Galβ1-4Glc-CL
**ABH16**	Galα1-3(Fucα1-2)Galβ1-3GlcNAc-CL
**ABH17**	Galα1-3(Fucα1-2)Galβ1-3GlcNAcβ1-3Gal-CL
**ABH18**	Galα1-3(Fucα1-2)Galβ1-3GlcNAcβ1-3Galβ1-4Glc-CL

**ABH19**	Galα1-3(Fucα1-2)Galβ1-4GlcNAc-CL
**ABH20**	Galα1-3(Fucα1-2)Galβ1-4GlcNAcβ1-3Gal-CL
**ABH21**	Galα1-3(Fucα1-2)Galβ1-3GalNAcβ1-3Gal-CL
**GA01**	Galβ1-3GalNAcβ1-4(Neu5Acα2-3)Galβ1-4Glc-CL
**GA02**	Fucα1-2Galβ1-3GalNAcβ1-4(Neu5Acα2-3)Galβ1-4Glc-CL
**GA03**	Neu5Acα2-3Galβ1-3GalNAcβ1-4Galβ1-4Glc-CL
**GA04**	GalNAcβ1-4Galβ1-4Glc-CL
**GA05**	Galβ1-3GalNAcβ1-4Galβ1-4Glc-CL
**GA06**	Neu5Aca2-8Neu5Aca2-3Galβ1-4Glc-CL
**GA07**	GalNAcβ1-4(Neu5Acα2-3)Galβ1-4Glc-CL
**GA08**	Galβ1-4(Neu5Acα2-3)Galβ1-4Glc-CL
**GA09**	Galβ1-3GalNAcβ1-4(Neu5Acα2-8Neu5Acα2-3)Galβ1-4Glc-CL
**GA10**	GalNAcβ1-4(Neu5Acα2-8 Neu5Acα2-3)Galβ1-4Glc-CL
**GL01**	Galα1-4Galβ1-4GlcNAc-CL
**GL02**	GalNAcβ1-3Galα1-4Galβ1-4Glc-CL
**GL03**	Galβ1-3GalNAcβ1-3Galα1-4Galβ1-4Glc-CL
**GL04**	GalNAcα1-3GalNAcβ1-3Gal-CL
**GL05**	GalNAcα1-3GalNAcβ1-3Galα1-4Galβ1-4Glc-CL
**GL06**	Galα1-4Galβ1-4Glc-CL
**GL07**	Neu5Acα2-3Galα-4Galβ-4Glc-CL
**GL08**	Neu5Acα2-3Galβ1-3GalNAcβ1-3Galα1-4Galβ1-4Glc-CL
**GL09**	Fucα1-2Galβ1-3GalNAcβ1-3Galα1-4Galβ1-4Glc-CL
**GL10**	GalNAcα1-3(Fucα1-2)Galβ1-3GalNAcβ1-3Galα1-4Galβ1-4Glc-CL

**GL11**	Galα1-3(Fucα1-2)Galβ1-3GalNAcβ1-3Galα1-4Galβ1-4Glc-CL
**HMO01**	Galβ1-4Glc-CL
**HMO02**	Galβ1-3GlcNAcβ1-3Galβ1-4Glc-CL
**HMO03**	Neu5Acα2-3Galβ1-4Glc-CL
**HMO04**	Fucα1-2Galβ1-4(Fucα1-2)Glc-CL
**HMO05**	Fucα1-2Galβ1-4Glc-CL
**HMO06**	Galβ1-4(Fucα1-3)Glc-CL
**HMO07**	Galβ1-4GlcNAcβ1-3Galβ1-4Glc-CL
**HMO08**	Neu5Acα2-6Galβ1-4Glc-CL
**HMO09**	GlcAβ1-3Galβ1-3GlcNAcβ1-3Galβ1-4Glc-CL
**LE01**	Galβ1-3(Fucα1-4)GlcNAc-CL
**LE02**	Galβ1-3(Fucα1-4)GlcNAcβ1-3Gal-CL
**LE03**	Galβ1-3(Fucα1-4)GlcNAcβ1-3Galβ1-4(Fucα1-3)Glc-CL
**LE04**	Fucα1-2Galβ1-3(Fucα1-4)GlcNAc-CL
**LE05**	Fucα1-2Galβ1-3(Fucα1-4)GlcNAcβ1-3Gal-CL
**LE06**	Galβ1-4(Fucα1-3)GlcNAc-CL
**LE07**	Galβ1-4(Fucα1-3)GlcNAcβ1-3Gal-CL
**LE08**	Neu5Acα2-3Galβ1-4(Fucα1-3)GlcNAcβ1-3Gal-CL
**LE09**	Galβ1-4(Fucα1-3)GlcNAcβ1-3Galβ1-4(Fucα1-3)Glc-CL
**LE10**	Fucα1-2Galβ1-4(Fucα1-3)GlcNAcβ1-3Gal-CL
**LE11**	Fucα1-2Galβ1-4(Fucα1-3)GlcNAc-CL
**P01**	Glcα1-4Glc-CL
**P02**	Glcβ1-4Glc-CL

P03 Galα1-6Glc-CL

P04 Glcα1-6Glcα1-6Glc-CL

Supplementary Table 2. Affinities (K_d) of OS^{mod} for $^{Uni}P_{proxy}$ measured by ESI-MS.[a,b]

OS^{mod}	K_d (μM)	OS^{mod}	K_d (μM)
**ABH01**	11.9 ± 0.7	**GA01**	10.3 ± 3.5
**ABH02**	16.4 ± 1.8	**GA10**	14.1 ± 1.9
**ABH03**	9.9 ± 3.2	**GA02**	18.7 ± 1.2
**ABH04**	9.2 ± 2.6	**GA03**	5.5 ± 1.7
**ABH05**	12.7 ± 3.8	**GA04**	10.8 ± 2.4
**ABH06**	4.1 ± 0.2	**GA05**	7.9 ± 1.0
**ABH07**	12.1 ± 2.4	**GA06**	26.4 ± 0.8
**ABH08**	18.4 ± 2.2	**GA07**	6.5 ± 0.3
**ABH09**	19.4 ± 2.9	**GA08**	12.4 ± 2.6
**ABH10**	15.8 ± 2.5	**GA09**	32.7 ± 1.2
**ABH11**	15.2 ± 0.8	**GL01**	10.4 ± 0.4
**ABH12**	22.4 ± 6.9	**GL02**	7.6 ± 1.5
**ABH13**	11.5 ± 3.0	**GL03**	7.4 ± 0.7
**ABH14**	4.7 ± 0.8	**GL04**	8.6 ± 0.9
**ABH15**	8.6 ± 3.7	**GL05**	6.9 ± 0.2
**ABH16**	32.9 ± 8.3	**GL06**	4.2 ± 0.8
**ABH17**	8.8 ± 2.2	**GL07**	14.0 ± 1.3
**ABH18**	16.0 ± 2.8	**GL08**	10.3 ± 1.3
**ABH19**	10.6 ± 2.4	**GL09**	18.1 ± 2.1
**ABH20**	13.0 ± 4.7	**GL10**	4.7 ± 0.4
**ABH21**	4.1 ± 0.8	**GL11**	7.9 ± 0.7
**HMO01**	5.1 ± 1.4	**LE04**	48.5 ± 5.0

**OS^{mod}**	**K_d (μM)**	**OS^{mod}**	**K_d (μM)**
**HMO02**	6.6 ± 0.7	**LE05**	40.2 ± 13.0
**HMO03**	4.0 ± 0.7	**LE06**	13.0 ± 4.4
**HMO04**	6.0 ± 0.2	**LE07**	10.4 ± 2.6
**HMO05**	12.1 ± 0.9	**LE08**	11.6 ± 4.6
**HMO06**	7.2 ± 1.2	**LE09**	43.3 ± 12.7
**HMO07**	2.2 ± 0.5	**LE10**	9.0 ± 2.8
**HMO08**	14.6 ± 2.1	**LE11**	8.5 ± 3.6
**HMO09**	10.6 ± 1.4	**P01**	1.7 ± 0.6
**LE01**	15.9 ± 3.4	**P02**	8.9 ± 1.5
**LE02**	11.9 ± 4.0	**P03**	6.6 ± 2.0
**LE03**	8.1 ± 2.7	**P04**	5.9 ± 0.3

a. Errors correspond to one standard deviation. b. Affinities measured in 200 mM aqueous ammonium acetate (pH 7, 25 ºC).

Supplementary Table 3. Affinities (K_d) of OS^{mod} for GBPs measured by CUPRA and affinities of the corresponding OS for the GBPs.[a,b]

GBP	OS^{mod}	T (ºC)	K_d (OS^{mod}) (μM)	K_d (OS) (μM)
SNA	**HMO08**	25	0.43 ± 0.01	NA [g]
MAA	**HMO03**	25	15 ± 1	NA [g]
MAA	**GA06**	25	8.3± 0.1	NA [g]
CTB_5	**GA01**	25	0.66 ± 0.01	0.31 ± 0.03 [c]
CTB_5	**GA02**	25	45 ± 4	135 ± 4
CBM51	**ABH11**	25	96 ± 10	14.3 ± 0.8 [d]
CBM51	**ABH12**	25	110 ± 10	38 ± 3
CBM51	**ABH13**	25	170 ± 40	40 ± 8
CBM51	**ABH15**	25	100 ± 10	17.2 ± 0.6 [d]
CBM51	**ABH19**	25	97 ± 18	17.2 ± 1.2 [d]
CBM51	**ABH20**	25	92 ± 12	38 ± 5
siglec-2	**HMO08**	25	110 ± 20	281 ± 10 [e,f]
siglec-2	**HMO08**	0	43 ± 10	NA [g]

a. Errors correspond to one standard deviation. b. Unless otherwise noted, affinities were measured in 200 mM aqueous ammonium acetate (pH 7) by ESI-MS. c. Values taken from Lin et al. *J. Am. Soc. Mass Spectrom.* **25**, 104–110 (2014). d. Values taken from Han et al. *Glycobiology* **27**, 170–180 (2015). e. Measured by ITC in aqueous solution (pH 8.0) of 20 mM Tris and 150 mM NaCl. f. Value taken from Ereño-Orbea, J. et al. *Nat. Commun.* **8**, 764 (2017). g. NA ≡ Not available.

Supplementary Table 4. List of MWs and saccharide compositions of extracted HMOs before and after introduction of CUPRA linker (CL).

HMO	MW (theoretical)	MW (experimental) [a]
Hex_2FucOH	488.17	488.18 ± 0.01
Hex_2Fuc-CL	1250.52	1250.51 ± 0.02
$Hex_2Neu5AcOH$	633.21	633.22 ± 0.01
$Hex_2Neu5Ac$-CL	1395.55	1395.55 ± 0.02
$Hex_3HexNAcOH$	707.25	707.26 ± 0.01
$Hex_3HexNAc$-CL	1469.59	1469.58 ± 0.02
$Hex_3HexNAcFucOH$	853.31	853.32 ± 0.01
$Hex_3HexNAcFuc$-CL	1615.65	1615.64 ± 0.02
$Hex_3HexNAcNeu5AcOH$	998.34	998.35 ± 0.01
$Hex_3HexNAcNeu5Ac$-CL	1760.68	1760.68 ± 0.03
$Hex_3HexNAcFuc_2OH$	999.36	999.37 ± 0.01
$Hex_3HexNAcFuc_2$-CL	1761.71	1761.70 ± 0.03
$Hex_4HexNAc_2FucOH$	1218.44	1218.45 ± 0.01
$Hex_4HexNAc_2Fuc$-CL	1980.78	1980.78 ± 0.03
$Hex_4HexNAc_2Fuc_2OH$	1364.50	1364.52 ± 0.01
$Hex_4HexNAc_2Fuc_2$-CL	2126.84	2126.82 ± 0.03
$Hex_4HexNAc_2Fuc_3OH$	1510.55	1510.56 ± 0.01
$Hex_4HexNAc_2Fuc_3$-CL	2272.90	2272.87 ± 0.04

a. Errors correspond to one standard deviation.

Supplementary Table 5. Apparent concentrations of modified (with CUPRA linker, CL) HMOs estimated from their binding to $^{Uni}P_{proxy}$ measured by ESI-MS and their apparent affinities (K_d, μM) to hGal-3C measured by CUPRA.[a,b]

HMO	Initial concentrations (μM) [c]	K_d (μM)
Hex_2Fuc-CL	9.0 ± 0.1	NB
$Hex_2Neu5Ac$-CL	8.4 ± 0.1	NB
$Hex_3HexNAc$-CL	15 ± 1	31 ± 4
$Hex_3HexNAcFuc$-CL	15 ± 1	2.5 ± 0.5
$Hex_3HexNAcNeu5Ac$-CL[d]	8.8 ± 0.2	20 ± 3
$Hex_3HexNAcFuc_2$-CL[d]	8.8 ± 0.2	20 ± 3
$Hex_4HexNAc_2Fuc$-CL	2.9 ± 0.1	29 ± 3
$Hex_4HexNAc_2Fuc_2$-CL	3.5 ± 0.1	87 ± 5
$Hex_4HexNAc_2Fuc_3$-CL	1.2 ± 0.1	NB

a. Errors correspond to one standard deviation. b. Affinities measured in 200 mM aqueous ammonium acetate (pH 7, 25 ºC). c. Values represent apparent concentrations corresponding to all HMOs of same MW (isomer set). d. Due to the small difference in MW, these two HMO isomer sets were treated as a single set.

Supplementary Table 6. Relative activities of sialylated OS^{mod} substrates measured by time-resolved CUPRA for NEU2 and NEU3 in 200 mM aqueous ammonium acetate (pH 7, 25 ºC).

Substrate	NEU2	NEU3
**GA01**	0.000	0.008
**GA02**	0.000	0.000
**GA03**	0.524	0.551
**GA06**	0.443	0.298
**GA07**	0.000	0.009
**GA08**	0.000	0.034
**GA09**	0.000	0.247
**GA10**	0.000	0.292
**GL07**	0.000	0.462
**GL08**	0.918	0.523
**HMO03**	1.000	1.000
**HMO08**	0.004	0.275
**LE08**	0.188	0.494
